# Advance Care Planning in Nursing Homes – Improving the Communication Among Patient, Family, and Staff: Results From a Cluster Randomized Controlled Trial (COSMOS)

**DOI:** 10.3389/fpsyg.2018.02284

**Published:** 2018-12-04

**Authors:** Irene Aasmul, Bettina S. Husebo, Elizabeth L. Sampson, Elisabeth Flo

**Affiliations:** ^1^Department of Global Public Health and Primary Care, Centre for Elderly and Nursing Home Medicine, University of Bergen, Bergen, Norway; ^2^Department of Nursing Home Medicine, Bergen, Norway; ^3^Division of Psychiatry, Marie Curie Palliative Care Research Department, University College London, London, United Kingdom; ^4^Department of Clinical Psychology, University of Bergen, Bergen, Norway

**Keywords:** advance care planning, dementia, nursing home, train-the-trainer, staff distress, COSMOS

## Abstract

**Introduction:** The majority of nursing home (NH) patients suffer from complex diseases, including dementia. This makes advance care planning (ACP) particularly important.

**Objectives:** The aim was to investigate the effect of an ACP intervention on communication among NH staff, patient, and family. We further investigated whether the intervention affected nursing staff distress.

**Methods:** The ACP intervention was a part of the 4-month cluster randomized controlled COSMOS trial with a 9-month follow-up. Norwegian NH units (*n* = 72), with 765 patients were invited, and eligible units were cluster randomized to usual care or the intervention group. The ACP intervention consisted of an education program targeting all NH staff (nurses and physicians) and managers. Implementation was supported by a train-the-trainer approach, with regular phone calls from the researchers. The effect of the intervention was assessed by a data collection form and questionnaires. Nursing staff distress was assessed by the Neuropsychiatric Inventory -Nursing Home version.

**Results:** Five hundred and forty five patients from 67 NH units were included and randomized to the intervention (*N* = 297; 36 units) and control group (*N* = 248; 31 units). Organized meetings between the family, patient, and nurses were conducted more frequently in the intervention compared to the control group at month 4 (OR = 3.9, 95% CI = 1.6 to 9.4, *p* = 0.002). Monthly contact between family and nurses was also more frequent in the intervention group (OR = 6.5, 95% CI = 1.6 to 3.5, *p* = 0.010). Nurses and families were more satisfied with their communication in the intervention compared to the control group. Staff distress was reduced in the intervention group at month 4 (*B* = -1.8, 95% CI = -3.1 to -0.4, *p* = 0.012). The intervention effect at month 4 did not persist during follow-up at month 9.

**Conclusion:** Compared to control, the ACP intervention improved the communication, and family and staff satisfaction as well as reduced staff distress. However, during the follow-up period these positive effects were not persistent. Indicating the necessity for ongoing staff support regarding ACP.

**Trial Registration:** www.ClinicalTrials.gov (NCT02238652).

## Introduction

The world’s population is aging rapidly (World Health Organization, 2018), and an increasing number of individuals are placed and ultimately die in nursing homes (NHs) (Hall et al., 2011). The majority of NH patients have dementia and multimorbidity is common (Gordon et al., 2014; Helvik et al., 2015). This leads to challenges in treatment and care, as the patients often have difficulties in expressing their individual needs (Selbaek et al., 2007).

Advance care planning (ACP) is an ongoing process of communication between healthcare providers, the patient and the family to clarify their understanding, wishes, values, and potential concerns about treatment and care at the end of life (Detering et al., 2010; Flo et al., 2016; Rietjens et al., 2017). Because cognitive decline and physical deterioration are difficult to predict, it is advisable that healthcare providers start the communication process even before NH admission when the person may still have capacity to make decisions for themselves (van der Steen et al., 2014; Martin et al., 2016).

The ACP process varies between NHs and countries worldwide. Local, cultural, and legal premises are essential because they determine the form and content of the ACP process (Sharp et al., 2013; Flo et al., 2016; Gjerberg et al., 2017). In a recent publication, we describe a novel ACP intervention in NHs as part of the COSMOS trial. We found that our clearly defined roles and responsibilities among the staff facilitated implementation of ACP, as well as targeting engagement of the NH managers (Aasmul et al., 2017). In a current Irish feasibility study including 290 long-term care and community hospital patients, McGlade et al. (2017) reported that more than 50% completed an end-of-life care plan, despite the reluctance of some nurses to participate in the ACP process because they thought of it as the responsibility of the NH managers (McGlade et al., 2017). In another study, Brazil et al. (2018) showed that uncertainty in decision-making related to patient care was reduced among the families who met with an ACP facilitator and received information about end-of-life care by mail (Brazil et al., 2018). Though family involvement is essential, previous studies have shown that patients and relatives rely on health personnel to initiate this type of communication (Fosse et al., 2014).

It is essential to educate the staff and create awareness of the need for ACP, and how the process should be conducted (Lacey, 2005). Today, the level of nursing staff competence varies, and training opportunities are scarce (Bing-Jonsson et al., 2016). Increasing complexity of patients’ conditions along with tougher job demands may lead to a lack of competence, and subsequent feelings of hopelessness and distress (Gautun and Syse, 2013). Consequently, education may reduce the gap between the nursing staffs’ competence and job demands and potentially also reduce staff distress (Sprangers et al., 2015).

Even though there has been an increased number of studies focusing on ACP, there is a need for well-powered RCTs that explore the communication in NHs, while also exploring the association with staff distress. Thus, the main objective in this paper was to investigate the effect of ACP on the communication among the NH staff, patient and family and whether the ACP intervention ameliorates staff distress. In particular, we hypothesized that the ACP intervention would:

• Improve the communication among staff, patients, and families;
• Increase the satisfaction with communication between the family and staff;
• Decrease nursing staff distress in the intervention compared to the control group.

## Methods

### Study Design

The ACP intervention was a part of the multicomponent, cluster randomized controlled COSMOS trial. The COSMOS acronym refers to each of the intervention components: ***Co***mmunication (in the form of ACP), ***S***ystematic pain assessment and treatment, ***M***edication review, ***O***rganization of activities and ***S***afety. Detailed information on the design, procedure, randomization and sample size analysis is described in the published protocol (Husebo et al., 2015). In brief, the calculation of sample size was based on change in the total score of the Neuropsychiatric Inventory -Nursing Home (NPI-NH) version. It was estimated that 520 patients from 64 NH units (clusters), would yield an 80% power to detect a 25% decrease in the NPI-NH total score in the intervention group compared to the control group, with a significance level of 5%. Eligible NH units were randomized to the intervention groups or care as usual (control groups) in each of the included municipalities. The randomization procedure was constrained to ensure that the intervention or control distribution was approximately equal matched to urban and rural, and prosperous and less well-to-do status.

### Participants and Settings

We invited eight municipalities from three counties in Southern Norway to participate. These included 37 NHs with 72 units and 765 patients. To achieve a representative sample, rural and urban, rich and poor municipalities were invited. The study was performed from August 2014 to December 2015.

Patients both with and without dementia were eligible to participate if they were >65 years, and had a minimum stay of 2 weeks in the NH before assessment. Exclusion criteria were: life expectancy < 6 months and schizophrenia. The intervention lasted 4 months, with assessments and data collection performed at baseline and month 4, additional follow-up assessments at month 9 were conducted to evaluate long-term effects of the intervention.

### Study Intervention

In a recent publication, we provide a detailed description of the content of the ACP intervention and evaluation of the implementation process in connection with the COSMOS trial (Aasmul et al., 2017). All registered and licensed practical nurses, physicians, and NH managers were invited to a 2-day education seminar. At least two nurses from each intervention unit were obliged to participate and were appointed COSMOS ambassadors. The nurses were given responsibilities in implementing the intervention in the units and in reporting progress to the researchers. The seminar included lectures, training, and role-play. The ACP education program was founded on evidence-based knowledge about ACP (Dening et al., 2011; Norwegian Directorate of Health, 2013; Flo et al., 2016). Amongst others, the ambassadors were introduced to the definition and perspectives of ACP. They were trained in how to involve family and initiate the communication process while remaining aware of question formulation: open-ended versus closed-ended questions and attentive listening. In relation to this, essential themes formulated as seven key questions were disseminated as flashcards the staff could carry in their uniform pockets, described and illustrated in our previous publication (Aasmul et al., 2017). The ambassadors were given a thorough presentation of the implementation material to be used back in the units, as the implementation relied on a train-the-trainer strategy (Orfaly et al., 2005). The COSMOS intervention included clearly defined tasks that should be performed by either staff or physician (COSMOS deliverables): providing an invitation to the patient and family to have a conversation with the physician and/or the primary nurse. Communication between the patient’s primary nurse and the family should be maintained monthly, and the family was also offered to be contacted by phone regularly by the primary nurse (phone calls could be replaced by occasional talks at the NH unit). Formal meetings including patient, family, primary nurse, and preferably the physician should be organized quarterly. Any stated preferences should be documented. To support implementation, the researchers followed up the ambassadors with phone calls every other week, and with discussions during a 1-day midway seminar comprising repetition and troubleshooting sessions.

### Assessments and Outcome Measures

Patient demographics including age and gender were extracted from the medical records. Cognitive function was assessed using the Mini Mental Status Examination (MMSE). It produces a sum score ranging from 0 to 30 used to follow the course of patients or to indicate the presence of cognitive impairment using cutoff scores, i.e., points ≥ 26 = no/questionable impairment, 21–25 = mild impairment, 11–20 = moderate, and 0 –10 = severe impairment (Folstein et al., 1975; Perneczky et al., 2006). The MMSE has been used extensively in clinical and research settings and has high test–retest reliability, internal consistency, and inter-rater reliability (Folstein et al., 1975; Velayudhan et al., 2014).

The NH managers documented whether the unit had participated in organized efforts to improve communication procedures during the past 3 years. Demographic data on the nursing staff were collected using short paper forms, during data collection at each unit.

The nursing staff used a data collection form in both the intervention and the control group to document the communication activities for each patient. As shown in Table 1, this form listed five different topics of communication deliverables: (a) Conversation with the NH physician, (b) Conversation with the patient’s primary nurse, (c) Monthly phone calls to the family, (d) Contact with the family the last month, and (e) Documented communication activities. The response options were yes, no, not applicable, and don’t know. In the statistical analyses, the “not applicable” option was combined with the “no” category and the “don’t know” option were set to missing.

**Table 1:** Concrete communication deliverables with patient and/or family^∗^.

Questions regarding types of communication deliverables the last month?
Have the patient and families been provided with an invitation to have a conversation with the physician?
Have the patient and families had a shared conversation with the primary nurse?
Have there been monthly phone calls to the family?
Have you had contact with the family during the past month?
Has the communication been documented?

^∗^*An overview of the registration of communication types at baseline, month 4 and month 9*.

To avoid situations where difficult subjects were forced on patients or family members, we encourage staff to organize a communication process with repeated meetings quarterly and to have contact with the family on a monthly basis (telephone and/or talks in the unit) (Aasmul et al., 2017). The different communication types provided during the study period were compared by the intervention and control group.

Each unit, represented by one nurse was asked to fill in a survey to document their perceived changes in communication both with the family and the physician on a Likert scale adapted from “Clinical global impression of change” (CGIC) with scores from minus 5 (*much worse communication*) to plus 5 (*much better communication*) (Olin et al., 1996; Schneider et al., 1997). A similar survey was mailed to the patients’ family and legal guardians at month 4, investigating whether the family had perceived changes regarding the communication with the patient’s primary nurse and the NH physician. Answers were given on an identical Likert scale from minus 5 (*much worse communication*) to plus 5 (*much better communication*).

Nursing staff distress was investigated by the use of the distress scale in the NPI-NH version, also known as occupational disruptiveness scale for the NPI-NH (Cummings et al., 1994; Kaufer et al., 1998). The inventory is a 12-item *proxy-rated* instrument, addressing different neuropsychiatric symptoms in the patient, and *self-reported* distress of these symptoms for the staff (Cummings et al., 1994). The staff distress scale consists of six levels: ‘not at all distressing’ (0), ‘minimally distressing’ (1), ‘mildly distressing’ (2), ‘moderately distressing’ (3), ‘severely distressing’ (4), and ‘extremely distressing’ (5). This means that the NH staff assesses how emotionally distressing the patient’s behavior is for the staff and if it entails more occupational burden (Ballard et al., 1996; Kaufer et al., 1998; Wood et al., 1999). It produces a sum score ranging from 0 to 60.

### Statistical Analyses

Statistical analyses were performed using Stata version 14 and IBM SPSS version 23. Descriptive data including demographic data of the NH staff and different communication types were calculated showing means, percentages and response rates. Differences in groups at baseline were examined by independent samples *t*-tests for normally distributed continuous variables, Mann–Whitney *U*-test for non-normal distributed continuous and Pearson X^2^ tests for categorical variables.

To investigate the effect of ACP on the communication between the NH staff, patient, and family, we conducted separate mixed effect logistic analyses with each of the following communication topics (Table 1) as outcome variables: (a) Conversation with the NH physician, (b) Conversation with the patient’s primary nurse, (c) Monthly phone calls to the family, (d) Contact with the family the last month, and (e) Documented communication activities. Changes in the outcome measures from baseline to 4 and 9 months were estimated by mixed effect logistic regression models. We treated time as a categorical variable, and included fixed effects for time, intervention, and their interaction in the models. To account for clustering, the models were fitted with patient specific random intercepts and NH-unit specific random intercept if it improved fit. Model selections were based on likelihood ratio tests.

To investigate nurses’ and families’ experiences of change in communication, separate linear regressions with robust estimation of standard error were performed with perceived change related to communication with the physician and nurse as outcome variables, and the dichotomous variable intervention group or control group as predictor variable.

To analyze the effect of the intervention on staff distress, we used linear mixed effect models with restricted maximum likelihood estimation (REML). The outcome measure was the NPI-NH staff distress score. We treated time as a categorical variable, and included fixed effects for time, intervention, and their interaction in the models. To account for clustering we included random intercepts for both NH-unit effects and patient-level effects, and a NH-unit specific random slope for time. The significance level was set to 0.05.

### Ethics Statement

The Regional Committee for Medical and Health Research Ethics, West Norway, approved the study (2013/1765). Written and verbal information about the study was provided to the patient and their family. The assessment of capability was done by trained researchers and the patient’s health care providers. In patients lacking the ability to consent, presumed written consent was obtained from his or her legal guardian, usually a family member.

## Results

**FIGURE 1:**
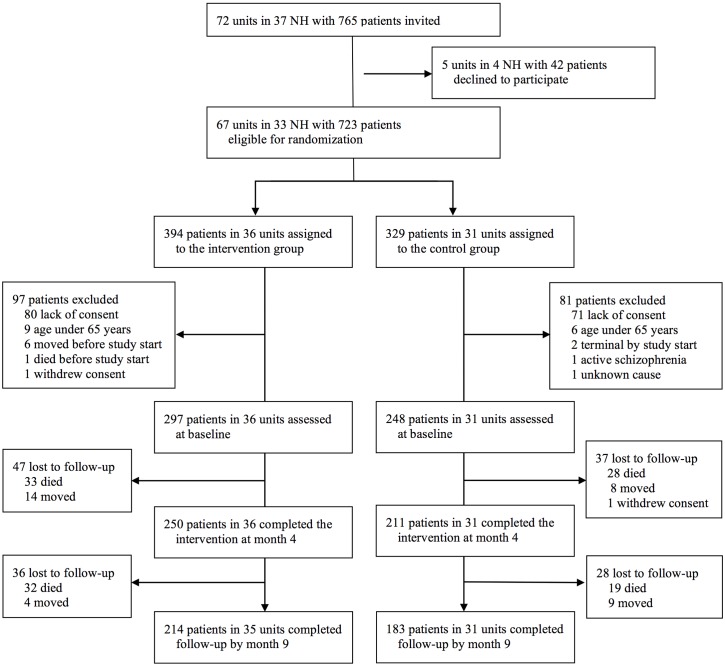
Flow-chart showing recruitment.

There were 5 units in 4 NHs with 42 patients that declined to participate, leaving 723 patients in 67 units from 33 NHs eligible for randomization. 36 units (394 patients) were randomized to the intervention group and 31 units (329 patients) to the control group (Figure 1). As explained in Figure 1, we excluded 97 patients from the intervention group and 81 from the control group, yielding 297 patients in the intervention (36 NH-units) and 248 patients in the control group (31 NH-units).

The included patients (*n* = 545) had a mean age of 87 years and 74% were women (Table 2). The mean MMSE score was 10.4 (*SD* = 7.6) in the intervention and 11.4 (*SD* = 7.9) in the control group. Of the total number of patients, 43% (*n* = 237) had severe cognitive impairment, and 3% (*n* = 18) of the patients had no or questionable cognitive impairment in accordance to the MMSE. At baseline, there were no significant differences between key patient characteristics (Table 2) or any of the other outcomes apart from more invitations to conversations with the NH physician reported in the intervention group (*n* = 28, 19%) compared to the control group (*n* = 15, 11%), *p* = 0.05 (Table 3). Between baseline and month 4, 13 patients were hospitalized in the intervention and 18 in the control group; this difference between groups was not significant.

**Table 2:** Patient characteristics and nursing staff distress at baseline.

NH patient characteristics:	Intervention (*n* = 297)	Control (*n* = 248)	*p*-Value for difference between groups^a^
Age, mean (*SD*)	87 (7.7)	87 (7.2)	0.40
Women, % (*n*)	73% (216)	75% (186)	0.55
**Cognitive function:**			
MMSE^b^ (0–30), Mean (SD)	10.4 (7.6)	11.4 (7.9)	0.17
No/questionable	9 (3%)	9 (4%)	-
(≥26), *n* (%)			
Mild (21–25), *n* (%)	21 (7%)	21 (9%)	-
Moderate (11–20), *n* (%)	107 (36%)	90 (36%)	-
Severe (0–10), *n* (%)	141 (47%)	96 (39%)	-
**Neuropsychiatric symptoms:**			
NPI-NH total NPS^c^ (0–144), mean (SD)	17.9 (19.6)	17.6 (20.7)	0.54
**Staff distress:**			
NPI-NH^d^ total staff distress (0–60), mean (SD)	7.3 (7.9)	6.8 (8.3)	0.33

*Data are presented as mean; *SD* = standard deviation; n = number of patients. The assessment scales range are in the brackets. ^*a*^Examined by independent samples *t*-tests for normally distributed continuous variables, Mann–Whitney *U*-test for non-normal distributed continuous variables and Pearson χ^*2*^ tests for categorical variables. ^*b*^MMSE = Mini-Mental Status Examination, higher score shows better cognitive function. ^*c*^NPI-NH total NPS = Neuropsychiatric Inventory- Nursing Home version -total Neuropsychiatric symptoms score, higher score shows more NPS. ^*d*^NPI-NH total staff distress = Neuropsychiatric Inventory -Nursing home version total staff distress, higher score shows more total staff distress*.

**Table 3:** Communication deliverables at baseline – month 4 and month 9.

	Baseline		Month 4		Month 9	
Communication types^a^:	Intervention *N* = 297 % (*n*)^b^	Control *N* = 248 % (*n*)^b^	Intervention *N* = 250 % (*n*)^b^	Control *N* = 211 % (*n*)^b^	Intervention *N* = 214 % (*n*)^b^	Control *N* = 183 % (*n*)^b^
Invitation to a conversation with the physician	19% (28)	11% (15)	18% (42)	15% (27)	21% (43)	15% (25)
	(RR = 49%)^c^	(RR = 56%)^c^	(RR = 96%)^c^	(RR = 84%)^c^	(RR = 96%)^c^	(RR = 95%)^c^
Shared conversation with primary nurse	28% (39)	29% (39)	38% (89)	19% (33)	35% (70)	25% (42)
	(RR = 46%)^c^	(RR = 45%)^c^	(RR = 93%)^c^	(RR = 81%)^c^	(RR = 94%)^c^	(RR = 93%)^c^
Monthly phone calls to the relatives	52% (72)	44% (59)	61% (147)	54% (89)	65% (133)	64% (108)
	(RR = 46%)^c^	(RR = 54)%^c^	(RR = 96%)^c^	(RR = 79%)^c^	(RR = 96%)^c^	(RR = 93%)^c^
Contact with the family the last month	90% (131)	91% (126)	93% (224)	84% (143)	93% (192)	90% (159)
	(RR = 49%)^c^	(RR = 56%)^c^	(RR = 96%)^c^	(RR = 81%)^c^	(RR = 97)%^c^	(RR = 97%)^c^
Documented communication	70% (88)	59% (69)	78% (176)	57% (89)	73% (138)	65% (102)
	(RR = 42%)^c^	(RR = 47%)^c^	(RR = 90%)^c^	(RR = 74%)^c^	(RR = 88%)^c^	(RR = 85%)^c^

^a^*The data collection form with different types of communication deliverables. ^*b*^% (*n*) = percent of, and number of patients answering “yes” to each of the listed communication deliverables. ^*c*^RR = response rate to each communication variable*.

As shown in Table 4 the nurses had an average of 17.5 years of working experience from the health sector, with 9 years from the current institution. None of the units had previously provided systematic education in ACP.

**Table 4:** Characteristics of the proxy raters (staff participating in the data collection) (*n* = 117)^1^.

Age, mean (SD)	42.3	(10.53)
Years of experience in health care, mean (SD)	17.5	(9.75)
Years of experience in this institution, mean (SD)	8.8	(6.65)
Female, *n* (%)	111	(95%)
**Nationality**		
Norwegian, *N* (%)	88	(75%)
Other European countries, *N* (%)	14	(12%)
South east Asia, *N* (%)	10	(9%)
Africa, *N* (%)	1	(1%)
**Education level**		
RN with additional education, *N* (%)	29	(25%)
RN	63	(54%)
LPN	23	(20%)
Other profession	2	(2%)

^1^*Due to missing values on some items, the sum of percentages is not 100. RN = registered nurse; LPN = licensed practical nurse*.

As shown in Table 5, the registration showed an increased number of the listed communication deliverables; “shared conversations between family, patient and the primary nurse” (OR = 3.9, 95% CI = 1.6 to 9.4, *p* = 0.002) and “contact with the families during the last month” (OR = 6.5, 95% CI = 1.6 to 3.5, *p* = 0.010) in the intervention group as compared to controls. There were no significant effects on the following communication deliverables; “Conversation with the physician,” “Monthly phone calls to the family,” or “Documentation of the communication.” Interestingly, none of the listed communication deliverables (Table 1) had a long-term effect at month 9.

**Table 5:** Change in communication and staff distress.

	Baseline to month 4			Baseline to month 9			ICC^a^ NH unit	ICC^b^ Patient
	Within-group change		Intervention effect	Within-group change		Intervention effect		
	Intervention	Control		Intervention	Control			
	OR (95% CI)^c^	OR (95% CI)^c^	OR (95% CI)^c^	OR (95% CI)^c^	OR (95% CI)^c^	OR (95% CI)^c^		
**Types of communication:**								
Invitation to a conversation with the physician	0.9 (0.5, 1.5)	1.5 (0.7, 3.0)	1.7 (0.7, 4.1)	1.1 (0.6, 2.0)	1.4 (0.7, 2.9)	1.2 (0.5, 3.1)	–	0.13
Shared communication with the primary nurse	1.6 (0.9, 2.9)	0.4** (0.2, 0.8)	3.9** (1.6, 9.4)	1.3 (0.7, 2.4)	0.6 (0.3, 1.1)	2.3 (0.9, 5.5)	0.33	0.39
Monthly phone calls to the family	1.4 (0.8, 2.5)	1.4 (0.8, 2.5)	1.0 (0.5, 2.2)	1.9** (0.4, 1.9)	2.5** (1.4, 4.4)	0.8 (0.3, 1.7)	0.19	0.39
Contact with the family the last month	1.5 (0.5, 4.0)	0.2** (0.8, 0.6)	6.5* (1.6, 3.5)	1.3 (0.5, 3.7)	0.5 (0.2, 1.4)	2.7 (0.6, 11.4)	0.18	0.68
Documented communication	1.5 (0.8, 2.8)	0.7 (0.4, 1.3)	2.1 (0.9, 4.8)	1.1 (0.6, 1.9)	1.1 (0.6, 2.0)	1.0 (0.4, 2.3)	0.32	0.38

	**B (95% CI)^d^**	**B (95% CI)^d^**	**B (95% CI)^d^**	**B (95% CI)^d^**	**B (95% CI)^d^**	**B (95% CI)^d^**		

**Total staff distress^e^**	-1.4** (-2.3, -0.5)	0.4 (-0.6, 1.4)	-1.8* (-3.1,-0.4)	-0.9 (-2.1, 0.3)	0.6 (-0.7, 1.9)	-1.5 (-3.3, 0.3)	0.19	0.62

*Both within and between group effects (intervention effect) from baseline to month 4 and from baseline to month 9 are provided for the entire study population. The staff reported whether the listed type of communication had been performed for each patient during the time period from baseline to month 4 and to month 9 (yes and no). ^*a*^Intracluster correlation on NH-units level. ^*b*^Intracluster correlation on patient level. ^*c*^Numbers indicate Odds Ratio for the topics of communication, where >1 equals an increase, and <a decrease. ^*d*^Numbers showing changes given by coefficient and 95% confidence interval. ^*e*^Staff distress assessed by Neuropsychiatric Inventory Nursing Home Version. ^∗^*p*-Value < 0.05; ^∗∗^*p*-value < 0.01*.

The nurses in the intervention group reported an improved communication with the patients’ families at month 4 compared to the controls (total response rate 55% *n* = 37/67, *B* = 1.9, 95% CI = 0.8 to 2.9, *p* = 0.001). Similarly, the families in the intervention group reported an improved satisfaction regarding the communication with the primary nurses (total response rate 67% *n* = 308/461, *B* = 0.4, 95% CI = 0.02 to 0.85, *p* = 0.040) at month 4, while no changes were found – neither in the families’ nor nurses’ experience of satisfaction concerning the communication with the NH physician (Table 6).

**Table 6:** Between-groups comparison regarding change in satisfaction with communication at month 4 among NH staff and family.

Communication assessed by:	Communication with family, B (95% CI)^a^	Communication with the primary nurse, B (95% CI)^a^	Communication with physician, B (95% CI)^a^
Nursing staff	1.9 (0.80, 2.91)^∗∗^		0.9 (-0.57, 2.37)
Family		0.4 (0.02, 0.85)^∗^	-0.1 (-0.47, 0.29)

^a^*Numbers showing the intervention effect by changes given by coefficient and 95% confidence interval. ^∗^p-value < 0.05; ^∗∗^p-value < 0.01*.

We found a reduction in nursing staff distress in the intervention as compared to the control group at month 4 (*B* = -1.8, 95% CI = -3.1 to -0.4, *p* = 0.012) assessed by NPI- NH distress scale (Table 5).

## Discussion

During the study period of 4 months, conversations between family, patient, and the primary nurse increased in the intervention group as compared to controls. An intervention effect was found regarding increased satisfaction with communication on the part of both the nurses and the family. In addition, there was a reduction in nursing staff distress. However, the effect did not persist at follow-up assessment at month 9.

The outcomes in this study are of high clinical relevance, as they indicate that the COSMOS ACP intervention changed NH routines (i.e., increased the number of meetings and conversations) and enhanced staff competence. The increased satisfaction suggests that these changes were perceived as positive for both family and staff. This study did not focus on the traditional outcomes when introducing ACP (e.g., number of “do not resuscitate” or “do not hospitalize” orders, feeding tube); consequently, it is difficult to compare the results of the present study with previous research in NHs.

This study included a very old and frail population, with a high prevalence of dementia. Although frailty and cognitive decline is common among NH patients (Clegg et al., 2013), research indicates that it is beneficial to include people with dementia in shared conversations (Allen et al., 2003; Dening et al., 2011). This was also an important message in our education program (Aasmul et al., 2017), as it is crucial, both from an ethical and clinical point of view, to include both the patient and family in the communication process (van der Steen et al., 2014).

The lack of effect at follow-up suggests that staff support is necessary to maintain a good routine for ACP in NHs. Initiating ACP is demanding on staff members, who are advised to start the process of ACP early, aiming to build up relationships by carefully considering timing and receptiveness for all the involved (van der Steen et al., 2014). This requires both staff skills, and well-established institutional routines that promote ACP meetings (Hickman et al., 2016; Aasmul et al., 2017; McGlade et al., 2017). Our education and follow-up helped the nursing staff to develop skills to initiate ACP, while the intervention’s focus on routines created a work setting that promoted such communication.

A previous study of the end-of-life care in Norwegian NHs, suggested that there is a need to involve the attending physicians and improve the communication abilities among staff (Gjerberg et al., 2011). In our study, we were successful in involving nurses, but found no increase in meetings with the physician or in satisfaction with communication with the physician. This might in part be because it was not mandatory for the physician to participate in the education seminar prior to the study. Indeed, Sharp et al. (2013) found that most physicians believed it was their professional responsibility to initiate discussions, but experienced difficulties achieving this due to limited time and lack of appropriate occasions (Sharp et al., 2013). Physicians also have limited formal training in end-of-life care, as part of their basic training (Gibbins et al., 2011; Fosse et al., 2017), and previous studies suggest a need for ACP education among physicians (Cavalieri et al., 2002; Dening et al., 2011).

We found a decrease in nursing staff distress in the intervention as compared to the control group. This is in line with previous studies, which have demonstrated a link between staff distress and staff competence in the NH setting (Aasmul et al., 2016). Increased knowledge may also empower staff members to cope with difficult symptoms (Hsu et al., 2007; Whitaker et al., 2014). The NPI-NH distress scale is associated with NPS (Wood et al., 1999; Selbaek et al., 2014). While this assessment is not optimal to uncover general staff distress, it is possible that the education and improved communication helped nursing staff to cope better with demanding symptoms of dementia. However, other variables which we had not encountered for, such as staff empathy, may have been a confounding variable. As discussed in a review by Wilkinson et al. (2017), there is evidence for a negative correlation between burnout and empathy (Wilkinson et al., 2017). Additionally, previous research suggests that other aspects such as organizational culture, the psychosocial environment and leadership affect both staff distress (Testad et al., 2010) and the implementation of ACP (Gilissen et al., 2017). To avoid potential disagreements in the NH units, the COSMOS ACP intervention aimed to clarify roles and responsibilities among staff, and involving the management. This may in turn have improved some aspects pertaining to the work environment.

This study suggests that the NH staff had difficulties continuing with ACP conversations when follow-up by researchers ended. The external facilitation is found to be key in improving outcomes in NHs (Seymour et al., 2011; Moore et al., 2017). The concept of ACP is complex, and support and guidance of the staff may be necessary to enable the units to maintain ACP conversations as part of the NH routine. We argue that there is no easy fix in this matter; as Bing-Jonsson et al. (2016) has shown, education, guidance and support of nursing staff is greatly needed in the NH setting (Bing-Jonsson et al., 2016). We have previously detected the importance of engaging NH managers along with the staff, working hands-on with patients as an important facilitator in implementing ACP (Aasmul et al., 2017). However, it may appear that this is not enough to change the NH culture over time. In the COSMOS ACP intervention, researchers telephoned the units every second week to discuss challenges and solutions. We suggest that this type of mentoring was an important part of successful implementation, along with continued attention to regular education in the units and a clear distribution of responsibilities (Aasmul et al., 2017).

### Strengths and Limitations

This study has a large sample size, with patients from different types of units, which promotes generalizability. The age and cognitive status in our study population reflects today’s NH population, ensuring ecological validity. To achieve implementation of a multicomponent intervention, staff follow-up was constantly optimized during the process. The number of patients participating in the shared conversations were not registered particularly, however staff were encouraged to act with attention to timing and sensitivity toward the patient and family’s current situation and understanding of the patient’s health status. The researchers’ close follow-up by both phone and written material allowed for a thorough evaluation of the ACP process and satisfaction among nursing staff and families. Even though, this was a cluster randomized controlled study, double-blinding was not possible. Instead, we maintained a single-blinded design, where the staff, patients, and family were not informed about group allocation. The staff, however, could deduce their group allocation if they were interested, because the NH management received information about the study, prior to the intervention.

The COSMOS trial combines several components in a multicomponent intervention. The complex design makes it more difficult to recognize whether we are measuring only the effect of ACP, or the effects of the combined components. However, we believe that the combination of these components represents the core level of care and treatment that should be expected in a modern care facility. By providing the multicomponent intervention, we ensured a minimum level of quality including ACP. It has been suggested that the extra attention from the staff (e.g., regular phone calls, discussions) contributes to increasing satisfaction among families, even if it does not measure the effect of ACP directly, it is a useful additional attribution (Sampson et al., 2011).

### Implications for Clinicians and Policy Makers

This study demonstrates that nursing staff education and follow-up improves communication about care and treatment and increases satisfaction. Our results are of high importance to both policymakers and NH managers, as they demonstrate benefits of increasing competence in nursing staff. Education with ongoing support should be a priority, to ensure that we meet the needs of the NH population. The frailty and impaired cognitive function in the study population illustrates the need to educate and empower healthcare professionals to initiate the communication process early, preferably while the patient still has the capacity to make informed decisions. Importantly, a well-established ACP routine in NHs appears to require a close staff follow-up and a continued focus on education. Thus, it is timely to highlight the need for a standard of care, ensuring that NHs provide qualified ACP.

## Conclusion

The study improved the frequency of communication and the satisfaction with communication among the patients’ families and the nurses. Additionally, nursing staff distress was reduced in the intervention group. This might be related to the focus on staff knowledge and enhanced competence provided by the intervention. The intervention effect did not persist beyond the intervention period; thus, we suggest that sustaining ACP necessitated close follow-up and staff support.

## Author Contributions

BH developed the study idea and applied for funding. BH and EF designed the study protocol, while IA had a main role in the data collection. IA drafted the first version of the manuscript with supervision from BH and EF. Contribution to the subsequent drafts were provided by IA, BH, ES, and EF. All authors approved the final version of the manuscript.

## Conflict of Interest Statement

The authors declare that the research was conducted in the absence of any commercial or financial relationships that could be construed as a potential conflict of interest.
